# Impact of Three-Month Androgen Deprivation Therapy on [68Ga]Ga-PSMA-11 PET/CT Indices in Men with Advanced Prostate Cancer—Results from a Pilot Prospective Study

**DOI:** 10.3390/cancers14051329

**Published:** 2022-03-04

**Authors:** Jing-Ren Tseng, Szu-Han Chang, Yao-Yu Wu, Kang-Hsing Fan, Kai-Jie Yu, Lan-Yan Yang, Ing-Tsung Hsiao, Feng-Yuan Liu, See-Tong Pang

**Affiliations:** 1 Department of Nuclear Medicine, New Taipei Municipal TuCheng Hospital (Built and Operated by Chang Gung Medical Foundation), New Taipei City 236, Taiwan; b9105019@cgmh.org.tw; 2 School of Medicine, Chang Gung University, Taoyuan 333, Taiwan; 3 Center for Advanced Molecular Imaging and Translation, Department of Nuclear Medicine, Linkou Chang Gung Memorial Hospital, Taoyuan 333, Taiwan; hans0406tammy@gmail.com (S.-H.C.); lp97ing@gmail.com (I.-T.H.); 4 Department of Radiation Oncology, Linkou Chang Gung Memorial Hospital, Taoyuan 333, Taiwan; shrinkiepig@gmail.com; 5 Department of Radiation Oncology, New Taipei Municipal TuCheng Hospital (Built and Operated by Chang Gung Medical Foundation), New Taipei City 236, Taiwan; khs.fan@gmail.com; 6 Division of Urology, Department of Surgery, Linkou Chang Gung Memorial Hospital, Taoyuan 333, Taiwan; cgurjay@gmail.com; 7 Biostatistics Unit, Clinical Trial Center, Linkou Chang Gung Memorial Hospital, Taoyuan 333, Taiwan; lyyang0111@gmail.com; 8 Department of Medical Imaging and Radiological Science, College of Medicine, Chang Gung University, Taoyuan 333, Taiwan

**Keywords:** prostate cancer, androgen deprivation therapy, [68Ga]Ga-PSMA-11 PET/CT imaging

## Abstract

**Simple Summary:**

Positron emission tomography/computed tomography (PET/CT) with prostate-specific membrane antigen (PSMA)-specific tracers is gaining traction for prostate cancer imaging. The aim of this pilot study was to evaluate PSMA PET/CT imaging response in 30 patients who had undergone three months of androgen deprivation therapy (ADT). Different imaging indices and the modified PET response criteria in solid tumors 1.0 served as outcome measures. Most patients showed a consistent reduction of PSMA PET/CT indices over three months. A total of 24 (80%) participants were partial responders. Patients in the International Society of Urological Pathology grade group 5 (*n* = 16) showed a less prominent reduction of the imaging indices, and none of them reached a complete response. Collectively, our data support the clinical usefulness of PSMA PET/CT imaging for monitoring treatment response after the first three months of ADT.

**Abstract:**

Purpose: The purpose of this pilot prospective study is to examine the gallium-68-prostate-specific membrane antigen-11 ([68Ga]Ga-PSMA-11) positron emission tomography/computed tomography (PET/CT) imaging response in patients with advanced or metastatic hormone-naïve prostate cancer (PC) after 3 months of androgen deprivation therapy (ADT). Methods: We prospectively included men with untreated, clinical stage III or IV PC scheduled to receive ADT for at least 6 months. [68Ga]Ga-PSMA-11 PET/CT images were obtained before the start of ADT and 10–14 weeks thereafter. The following indices were examined: maximum standardized uptake value (SUVmax), mean SUV, PSMA total volume, and PSMA total lesion values of the prostate, nodes, bones, and whole-body. The therapeutic response was assessed using the modified PET response criteria in solid tumors 1.0. A subgroup analysis of patients with the International Society of Urological Pathology (ISUP) grade group 5 versus <5 was also performed. Results: A total of 30 patients were eligible. All PSMA PET/CT indices were significantly reduced (*p* < 0.001) after 3 months of ADT. Twenty-four (80%) patients showed partial response. Complete response, stable disease, and disease progression were observed in two patients each. Sixteen patients with ISUP grade group 5 showed a less prominent SUVmax reduction (*p* = 0.006), and none of them reached complete response. Conclusions: Three months of ADT in patients with untreated, advanced PC significantly reduced PSMA PET/CT indices. While most participants partially responded to ADT, patients with ISUP grade group 5 showed a less prominent SUVmax reduction. Collectively, our pilot results indicate that [68Ga]Ga-PSMA-11 PET/CT imaging holds promise to monitor treatment response after the first three months of ADT.

## 1. Introduction

Despite decades of research, prostate cancer (PC) remains a significant public health concern among men [1]. Androgen deprivation therapy (ADT) resulting in testosterone suppression has been traditionally considered the standard of care for hormone-sensitive advanced or metastatic PC, although novel therapeutic options (e.g., docetaxel, abiraterone, enzalutamide, or apalutamide) hold promise for further improving outcomes [2].

Prostate-specific membrane antigen (PSMA) is highly expressed in PC and has theranostic applications [3]. Positron emission tomography/computed tomography (PET/CT) with PSMA-specific tracers is effective for imaging the prostate, lymph nodes, and distant sites in patients with PC [4]. PSMA PET/CT outperforms conventional imaging (i.e., CT and bone scanning) in terms of per-patient sensitivity for primary staging of PC, without significant differences in terms of specificity for the detection of regional metastases [5]. In addition, PSMA-based PET/CT is superior in detecting distant metastases [5].

An increased PSMA expression in PC cells activates signaling pathways that promote tumor cell survival and proliferation [6]. Interestingly, androgen deprivation enhances PSMA expression in both androgen-sensitive and castration-resistant PC cells [7]. Furthermore, an animal study demonstrated that both enzalutamide administration and orchidectomy can enhance PSMA expression in mice harboring xenografts expressing PSMA [8]. Collectively, these data indicate that the use of ADT might affect PSMA PET/CT findings in patients with PC [9,10]. However, published clinical data in this area are limited and inconclusive [11], and the impact of the time interval between the start of ADT and imaging has not been adequately taken into account [12]. In addition, a study in patients with biochemical recurrence has shown that ADT may increase the number of identifiable lesions on PSMA-based PET/CT images [13]. The aim of this pilot prospective study is to examine the PSMA PET/CT response after three months of ADT in patients with advanced or metastatic hormone-naïve PC.

## 2. Materials and Methods

### 2.1. Ethics Statement

The study was registered in ClinicalTrials.gov (identifier: NCT03977610), posted on 6 June 2019. The research protocol was approved by the Institutional Review Board of Chang Gung Memorial Hospital at Linkou, Taoyuan, Taiwan (approval number: 201801384A0) and all participants provided written informed consent. Data utilized in the analysis were anonymized, and personal information was removed.

### 2.2. Patient Selection

The inclusion criteria were as follows: (1) age between 40 and 85 years, (2) biopsy-proven diagnosis of prostate adenocarcinoma, (3) clinical stage III or IV, (4) no previous treatment, and (5) scheduled ADT given as monotherapy for at least 6 months. Patients who met at least one of the following criteria were excluded: (1) unwillingness to participate, (2) inability to comply with or tolerate [68Ga]Ga-PSMA-11 PET/CT imaging, (3) reduced glomerular filtration rate (<30 mL/min/1.73 m^2^), and (4) history of malignancy other than PC. The baseline [68Ga]Ga-PSMA-11 PET/CT scan was scheduled in the two weeks preceding the start of ADT. A follow-up examination was planned after 10–14 weeks of treatment.

### 2.3. [68Ga]Ga-PSMA-11 PET/CT Acquisition and Reconstruction

[68Ga]Ga-PSMA-11 was produced as previously described [14] and a median tracer dose of 141 MBq (range, 103–182 MBq) was administered. At 60 min after the tracer injection, the patients were imaged on a GE Discovery MI PET/CT scanner (GE Healthcare, Milwaukee, WI, USA). No contrast media were used. CT imaging settings were as follows: 120 kVp, automatic mA selection (range: 30–300 mA), 40 × 0.625 detector collimation, and a pitch of 0.984. Transaxial PET images were reconstructed with a field of view of 700 mm, a matrix size of 256 × 256, and a slice thickness of 5 mm. PET images were acquired using an acquisition time of 3 min per single-bed position, with the acquisition proceeding from the thigh to the skull. The Bayesian penalized likelihood algorithm Q.Clear was used to reconstruct PET images (beta value: 550). Images were corrected by using the attenuation correction, the point spread function, and the QCHD-S technique.

### 2.4. [68Ga]Ga-PSMA-11 PET/CT Indices

Two expert nuclear medicine physicians (J.R.T. and F.Y.L.) independently reviewed all images and identified malignant PC lesions characterized by an increased [68Ga]Ga-PSMA-11 uptake. Any discrepancies were resolved through a consensus discussion. The PMOD 3.3 software (PMOD Technologies, Zurich, Switzerland) was used for manual delineation of volumes of interest on each lesion. The segmentation of soft tissue lesions was performed by applying a threshold for voxel standardized uptake values (SUVs) calculated according to a previously described formula [15]. The segmentation of bone lesions was undertaken using a fixed threshold of 3.0 [15]. Voxels characterized by SUV values above the threshold were considered malignant lesions. We calculated the following parameters: maximum SUV (SUVmax), mean SUV, and metabolic tumor volume (MTV). In line with a previous study [16], MTV was labeled as PSMA total volume (PSMA-TV). PSMA total lesion (PSMA-TL) was calculated by multiplying the mean SUV of each lesion and PSMA-TV. Whole-body SUVmax (wbSUVmax) was defined as the maximum SUVmax of all malignant lesions in each patient, whereas whole-body PSMA-TV (wbPSMA-TV) and whole-body PSMA-TL (wbPSMA-TL) were defined as the sum of PSMA-TV and PSMA-TL for all malignant lesions in each patient, respectively. The following imaging indices were examined for the purpose of analysis: SUVmax, PSMA-TV and PSMA-TL of prostate tumor, metastatic lymph nodes, and bone metastases, as well as whole-body indices (wbSUVmax, wbPSMA-TV, and wbPSMA-TL).

### 2.5. Definition of Response and Reduction Ratio

The modified PET response criteria in solid tumors (mPERCIST) [17] were used to assess the metabolic response before the start of ADT and 3 months thereafter. Complete response (CR) was defined as the disappearance of any lesion with tracer uptake. A partial response (PR) was considered present when a > 30% reduction in uptake value and tumor volume was observed. Progression of disease (PD) was defined as the appearance of at least two novel lesions or a ≥ 30% increase in tumor volume or uptake value. The remaining patients were considered as having stable disease. To evaluate the differences before and after ADT, the reduction ratio of an imaging index was defined as one minus the value measured after ADT divided by the value recorded before ADT. When the index showed a post-ADT increase, the reduction ratio became negative.

### 2.6. Subgrouping of Tumor Aggressiveness with The ISUP Grade Group and ADT Schemes

The classification of pathological tumor aggressiveness was in accordance with the recommendations of the International Society of Urological Pathology (ISUP) grade group [18]. Owing to the presence of advanced disease, the study participants were divided into two subgroups, as follows: patients with ISUP grade groups less than 5 versus patients with grade group 5. We also divided the study patients according to the ADT schemes, as follows: monotherapy with leuprorelin or goserelin versus combination therapy with leuprorelin, goserelin, or triptorelin, and other hormonal agents.

### 2.7. Data Analysis

Categorical variables are given as counts and percentages, whereas continuous data are expressed as means and standard deviations. Differences in imaging indices before and after ADT were assessed using the paired Wilcoxon signed-rank test. The associations between the reduction ratios of prostate-specific antigen (PSA) and various [68Ga]Ga-PSMA-11 PET/CT indices were evaluated using the Spearman’s rank correlation coefficient. The Mann–Whitney *U* test was used to examine the differences according to different ISUP grades. Analyses were performed using SPSS (version 28.0; IBM Corp., Armonk, NY, USA), with all tests two-sided at a 5% level of significance.

## 3. Results

### 3.1. Patient Characteristics and Therapy

**Figure 1:**
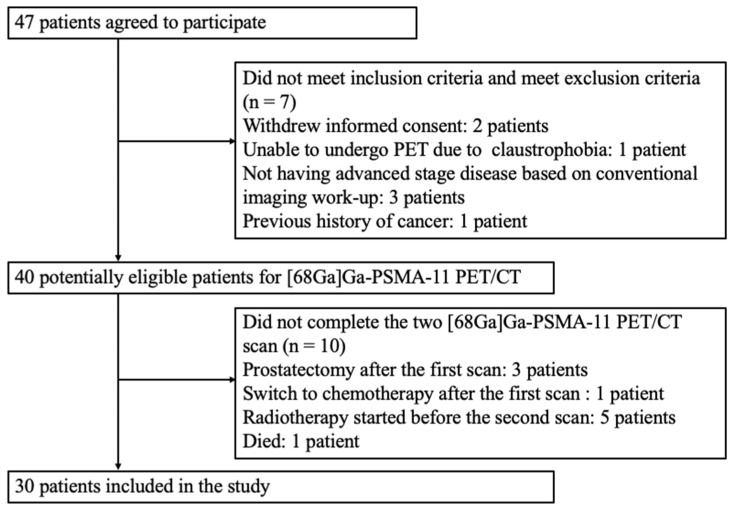
Flow of patients through the study.

Figure 1 depicts the flow of patients through the study. From October 2019 to April 2021, 47 patients scheduled for ADT were considered as candidates for the study. After appropriate exclusions, the final study cohort consisted of 30 patients who received either monthly or three-monthly ADT. The general characteristics of the study patients are shown in Table 1. Twenty-one patients (70%) had regional or distant metastases. According to the National Comprehensive Cancer Network Guidelines, the remaining 9 patients (30%) were classified in the high-risk group. No patient had a pathological diagnosis of neuroendocrine PC or ductal prostate adenocarcinoma.

### 3.2. Serum PSA Values and Reduction Ratios

The median duration of ADT before the second [68Ga]Ga-PSMA-11 PET/CT scan was 86 days (mean: 87 days; range: 80–104 days). Serum PSA values before and after ADT are presented in Table 2. The mean and median baseline serum PSA concentrations in the study participants were 117 and 42 ng/mL, respectively (range: 7.9−725.8 ng/mL). The mean and median serum PSA concentrations at the time of second scan were 4.5 and 0.67 ng/mL, respectively (range: 0.03−48.0 ng/mL). Compared to the baseline, a significant reduction in PSA levels was observed (mean reduction ratio: 0.95; range: 0.79−1.00, *p* < 0.001). We found no significant differences between ISUP subgroups, although mean PSA levels were lower in patients with ISUP grade groups < 5.

### 3.3. Baseline [68Ga]Ga-PSMA-11 PET/CT Imaging

The baseline [68Ga]Ga-PSMA-11 PET/CT findings are presented in Table 3. We identified one and four patients with nodal and bone disease spread, respectively, who were misclassified as negative on traditional imaging. A patient with suspected metastatic disease to a single lymph node on CT was considered as negative on [68Ga]Ga-PSMA-11 PET/CT. The measured [68Ga]Ga-PSMA-11 PET/CT indices are presented in Table 4.

### 3.4. [68Ga]Ga-PSMA-11 PET/CT Imaging after ADT

**Figure 2:**
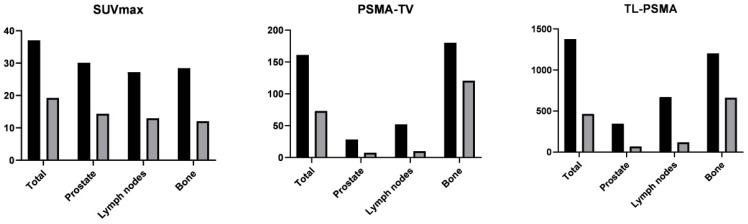
Mean [68Ga]Ga-PSMA-11 PET/CT indices before (black columns) and after (gray columns) androgen deprivation therapy. All indices decreased significantly. SUVmax, maximum standardized uptake value; PSMA-TV, prostate-specific membrane antigen—total volume; PSMA-TL, prostate-specific membrane antigen—total lesion.

The changes in [68Ga]Ga-PSMA-11 PET/CT indices after ADT are presented in Table 4 and Figure 2. The reduction ratios of SUVmax for the prostate tumor, metastatic lymph nodes, and metastatic bone lesions were 49%, 74%, and 63%, respectively, with those of PSMA-TV and PSMA-TL being 50%, 89%, and 65% and 70%, 92%, and 73%, respectively. The reduction was most prominent for metastatic lymph nodes, followed by metastatic bone lesions and the prostate tumor. We found no significant associations between the PSA reduction ratio and the reduction ratios of [68Ga]Ga-PSMA-11 PET/CT indices.

**Figure 3:**
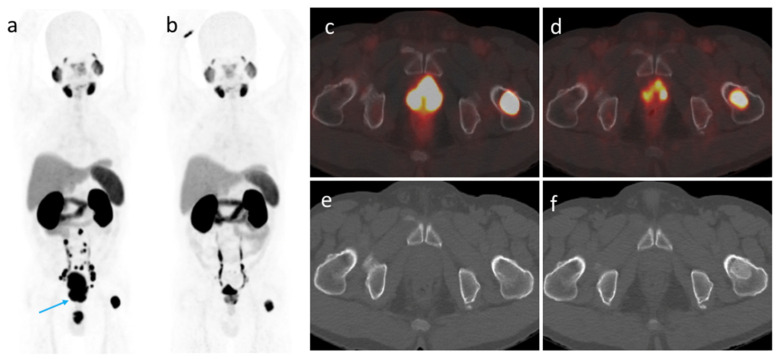
A 60-year-old patient with metastatic prostate cancer (clinical stage IVB, PSA 11.54 ng/mL, Gleason score 5 + 4). (**a**) A maximum intensity projection obtained before treatment revealed the prostate tumor (blue arrow) accompanied by urine contamination. (**b**) A second scan after three months of leuprorelin therapy showed a significant response to treatment. (**c**) and (**d**) Axial fused PET/CT images revealed a decreased prostate tumor metabolic volume and a reduced tracer uptake. The SUVmax of a left femoral metastatic lesion decreased from 70.41 to 20.32. (**e**) and (**f**) A second morphological imaging examination revealed a bony sclerotic change of the left femoral metastatic lesion, which was unexpected based on PET/CT findings.

According to the mPERCIST criteria, 2 patients (6.7%) were categorized as showing CR, 24 (80%) as PR, 2 (6.7%) as SD, and 2 (6.7%) as PD. Metastatic lymph nodes and bone lesions completely regressed in 6 (37.5%) out of 16 patients and in 5 (33.3%) out of 15 patients, respectively. An illustrative example of a patient who achieved PR was presented in Figure 3. The two patients who showed PD displayed increased primary tumor PSMA-TV values (from 1.42 to 5.99 cm^3^ and from 1.36 to 4.01 cm^3^, respectively). In addition, their serum PSA levels decreased from 8.8 to 0.38 ng/mL and from 47.9 to 1.38 ng/mL, respectively. On a related note, SUVmax decreased from 59.5 to 30.5 and from 90.2 to 85.6, respectively. The first patient had local disease only, whereas the second showed small nodal and bone metastases. Obvious regression of the metastatic lesions was observed after three months of ADT. Based on PSMA PET/CT findings, both patients underwent additional radiation therapy, which was followed by a significant PSA decrease (0.01 and 0.03 ng/mL, respectively).

### 3.5. Changes in [68Ga]Ga-PSMA-11 PET/CT Indices According to The ISUP Grade Groups and ADT Schemes

**Figure 4:**
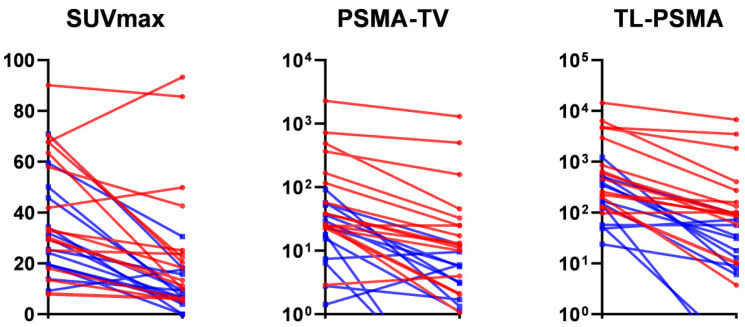
[68Ga]Ga-PSMA-11 PET/CT indices before and after androgen deprivation therapy. Red lines: Gleason group 5; blue lines: other Gleason groups. SUVmax, maximum standardized uptake value; PSMA-TV, prostate-specific membrane antigen—total volume; PSMA-TL, prostate-specific membrane antigen—total lesion.

A comparison of [68Ga]Ga-PSMA-11 PET/CT indices between ISUP subgroups is presented in Table 5. Patients with ISUP grade 5 showed a significantly lower wbSUVmax reduction ratio than those with grades less than 5 (0.35 versus 0.60, respectively, *p* = 0.006). Borderline significant differences in the reduction ratios of wbPSMA-TV and wbPSMA-TL were also observed. Both wbPSMA-TV and wbPSMA-TL were significantly higher in patients with ISUP grade 5 both before and after ADT. Figure 4 illustrates the changes of [68Ga]Ga-PSMA-11 PET/CT indices in different ISUP groups. [68Ga]Ga-PSMA-11 PET/CT indices did not differ significantly across different ADT schemes and the therapeutic approach was not found to affect the response (Table S1).

## 4. Discussion

This is, to our knowledge, the first prospective study to investigate [68Ga]Ga-PSMA-11 PET/CT indices after 3 months of ADT in patients with advanced or metastatic hormone-naïve PC. We found that both serum PSA levels and [68Ga]Ga-PSMA-11 PET/CT indices were significantly reduced at the time of the second scan. The reduction of PET/CT indices was most prominent for metastatic lymph nodes, followed by metastatic bone lesions, and the prostate tumor. On analyzing treatment response with the mPERCIST criteria, 24 patients (80%) showed PR, whereas CR, SD, and PD were identified in 2 patients each. Further longitudinal research is necessary to assess whether these findings have prognostic implications.

Previous in vitro studies have shown that PSMA expression increases in response to ADT [19,20,21]. It has been also suggested that ADT may induce flare phenomena [22]. Preclinical studies have reported a 2- to 4-fold increase in PSMA expression after an ADT of 1–4 weeks [11]. In an animal xenograft study [8], enzalutamide administration and castration decreased tumor size. However, the amount of PSMA expression per cell increased significantly in the post-ADT period. The inconsistencies in the published studies may at least in part originate from the opposite effects of ADT on tumor size and PSMA expression. Imaging investigations should yield reliable results in the short-term when ADT is expected to increase PSMA expression. However, long-term ADT may yield inconclusive imaging findings related to the volumetric reduction of PC [11]. The time interval from the start of ADT to the imaging scan is therefore critical. Onal et al. [23] have previously published a retrospective study which assessed the impact of neoadjuvant ADT on PSMA PET/CT findings in 108 patients with non-metastatic hormone-naïve PC. They found that both serum PSA and tumor SUV decreased significantly after a median interval of 2.9 months from the start of ADT. Approximately 15% of the study patients had evidence of progressive disease on PSMA PET/CT images, of whom half had their radiotherapy modified in light of these findings. Our study is the first to prospectively analyze the effect of ADT on PSMA PET/CT findings in patients with advanced or metastatic hormone-naïve PC.

Using serial [68Ga]Ga-PSMA-11 PET scans, Emmett et al. [24] reported a 30% reduction in SUVmax after nine days of treatment with luteinizing hormone-releasing hormone (either with or without bicalutamide) in eight men with metastatic hormone-sensitive PC. In another group of seven patients with castrate-resistant PC who received either enzalutamide or abiraterone, they showed a SUVmax increase of 45% after nine days. These authors suggested that, in the hormone-naïve stages of the disease, ADT may exert anti-proliferative effects which were reflected by a lower [68Ga]Ga-PSMA-11 uptake. In a prospective study conducted in nine patients with treatment-naïve PC, Ettala et al. [25] performed [68Ga]Ga-PSMA-11 PET/MRI imaging before degarelix-based ADT and three times thereafter. After 3–4 weeks of therapy, PSMA increased heterogeneously—especially in distant bone lesions. However, none of the metastatic lesions was found to disappear. In light of these inconsistencies, the optimal time point for [68Ga]Ga-PSMA-11 PET imaging after ADT has not been yet elucidated [26,27]. Our pilot results indicate that three months of ADT lead to significant changes in PSMA PET/CT indices. Therefore, this imaging modality is unsuitable for staging purposes within this timeframe.

A previous retrospective study analyzed 10 patients who received PSMA PET/CT imaging before and after continuous ADT [12]. A total of 31 lesions were visible in the pre-ADT phase. After a median of 230 days, the authors identified 14 lesions in eight patients. Additionally, a marked decrease (71%) in mean tracer uptake was evident [12]. In our study, two patients (7%) showed complete resolution of PC lesions after ADT. Hoberück et al. [28] conducted a retrospective study in 21 treatment-naïve patients with distant metastases who had undergone PSMA PET imaging before and after ADT (median duration: 155 days). Compared with the baseline, the results revealed a 24.0% and 13.9% decrease in wbPSMA-TV and wbPSMA-TL values, respectively. Another retrospective study [29] in 43 castration-naïve patients with PC showed that ADT induced a more significant SUVmax reduction in metastatic nodes compared to the primary tumor—a finding in line with our current observations.

Neoadjuvant ADT can decrease SUVmax values in patients with non-metastatic PC, with lower rates of metabolic response for cases with a Gleason score > 7 [23]. The results of subgroup analyses in our study revealed that patients with ISUP grade 5 showed a significantly lower SUVmax reduction ratio after ADT. On analyzing different ADT schemes, i.e., use of leuprorelin or goserelin as monotherapy versus combined use of leuprorelin, goserelin, and triptorelin with other hormonal agents, we found no significant intergroup differences in terms of PET/CT indices and treatment response. However, these results should be interpreted cautiously because of the small sample size.

**Figure 5:**
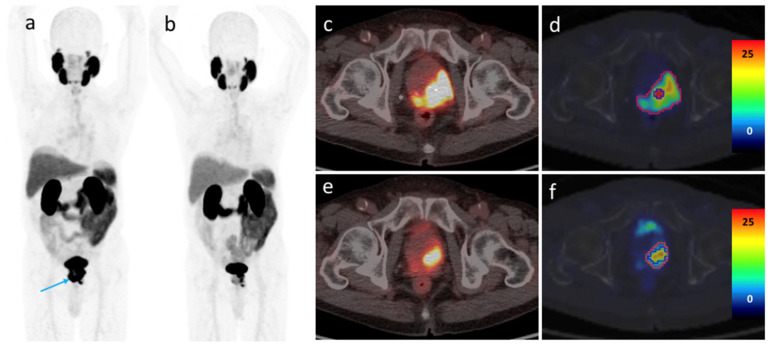
An 85-year-old patient with prostate cancer (clinical stage IVB, PSA 84.15 ng/mL, Gleason score 5 + 4). (**a**) A maximum intensity projection obtained before treatment revealed the prostate tumor (blue arrow) and a focal lesion beneath the primary lesion (pubic bone metastasis). (**b**) A second scan after three months of goserelin therapy showed a significantly decreased tumor metabolic volume. (**c**) and (**d**) Different tumor segmentation methods (threshold method: red contour; 45% SUVmax method: blue contour) yielded varying results. PSMA-TV and PSMA-TL values were 4.52 and 190.39, respectively (45% SUVmax method) and 53.59 and 824.69, respectively (threshold method). (**e**) and (**f**) After androgen deprivation therapy, PSMA-TV and PSMA-TL values were 3.14 and 44.87, respectively (45% SUVmax method) and 14.53 and 119.36, respectively (threshold method).

Different tumor segmentation methods may have an impact on [68Ga]Ga-PSMA-11 PET/CT indices. When the isocontor threshold was set at 45% of the lesion SUVmax, visual inspection showed an apparently inadequate tumor volume—especially in patients with high SUVmax [30]. An illustrative example of how this method could result in an underestimation of the primary tumor volume is shown in Figure 5. Therefore, this thresholding method was not applied in this study.

PSMA imaging may theoretically serve as a biomarker for assessing treatment response. However, this application may face significant challenges since PSMA avidity can be affected by several factors (e.g., castration sensitivity, timing and type of therapy, or lesion localization) [31]. According to the consensus statement on the PSMA PET/CT response criteria, patients with hormone-sensitive prostate cancer should not undergo this imaging modality within the first three months of systemic therapy [17].

There are limitations to this study. The small sample size and the heterogeneity of ADT regimens may have limited the validity of the findings, and, for that reason, larger prospective cohorts are required. More research is necessary to confirm our data and to evaluate the impact of specific therapeutic schemes and their duration on [68Ga]Ga-PSMA-11 PET/CT indices. Novel therapeutic options (e.g., docetaxel, abiraterone, enzalutamide, or apalutamide) hold promise for further improving outcomes in patients with metastatic hormone-sensitive PC. Unfortunately, financial constraints and lack of health insurance coverage have limited the use of these drugs in our study cohort. 

## 5. Conclusions

In conclusion, three months of ADT in patients with untreated, advanced PC significantly reduced PSMA PET/CT indices. While most participants partially responded to ADT, patients with ISUP grade group 5 showed a less prominent SUVmax reduction. Collectively, our pilot results indicate that [68Ga]Ga-PSMA-11 PET/CT imaging holds promise to monitor treatment response after the first three months of ADT.

## Figures and Tables

**Table 1:** Patient Characteristics and Differences between ISUP Subgroups.

	Number of Patients (%)			
Characteristic	Entire Cohort (*n* = 30)	ISUP Grade < 5 (*n* = 14)	ISUP Grade 5 (*n* = 16)	*p*
Age, years	69.8 ± 11.1	71.9 ± 9.8	68.1 ± 12.2	0.355
ECOG performance status				1.0
0	22 (73)	10 (71)	12 (75)	
1	8 (27)	4 (29)	4 (25)	
AJCC stage (8th edition)				0.068
IIIB	7 (23)	7 (50)	0 (0)	
IIIC	2 (7)	0 (0)	2 (12)	
IVA	6 (20)	2 (14)	4 (25)	
IVB	15 (50)	5 (36)	10 (63)	
Gleason score				<0.001
7	9 (30)	9 (64)	0 (0)	
8	5 (17)	5 (36)	0 (0)	
9	13 (43)	0 (0)	13 (81)	
10	3 (10)	0 (0)	3 (19)	
ISUP grade				<0.001
2	2 (7)	2 (14)	0 (0)	
3	7 (23)	7 (50)	0 (0)	
4	5 (17)	5 (36)	0 (0)	
5	16 (53)	0 (0)	16 (100)	
ADT regimen				0.289
Leuprorelin + bicalutamide	7 (23)	5 (36)	2 (12)	
Goserelin + bicalutamide	9 (30)	6 (42)	3 (19)	
Goserelin	4 (13)	1 (7)	3 (19)	
Leuprorelin	2 (7)	1 (7)	1 (6)	
Leuprorelin + cyproterone	3 (10)	1 (7)	2 (12)	
Leuprorelin + abiraterone	2 (7)	0 (0)	2 (12)	
Leuprorelin + abiraterone + bicalutamide	2 (7)	0 (0)	2 (12)	
Triptorelin + cyproterone	1 (3)	0 (0)	1 (6)	

ADT, androgen deprivation therapy; AJCC, American Joint Committee on Cancer; ECOG, Eastern Collaborative Oncology Group; ISUP, International Society of Urological Pathology.

**Table 2:** Serum PSA concentrations and differences between ISUP subgroups.

Characteristic	Entire Cohort (*n* = 30)	ISUP Grade < 5 (*n* = 14)	ISUP Grade 5 (*n* = 16)	*p*
Initial serum PSA, ng/mL	117 ± 200	59 ± 101	168 ± 250	0.208
Serum PSA at 2nd PET/CT scan, ng/mL	4.5 ± 12.0	1.3 ± 1.5	7.3 ± 15.6	0.240
Reduction ratio of serum PSA	0.95 ± 0.06	0.95 ± 0.07	0.96 ± 0.04	0.580

ISUP, International Society of Urological Pathology; PSA, prostate-specific antigen. Data are expressed as means ± standard deviations.

**Table 3:** Baseline [68Ga]Ga-PSMA-11 PET/CT findings and response assessment 3 months after ADT initiation.

Parameter	Entire Cohort (*n* = 30)	ISUP Grade < 5 (*n* = 14)	ISUP Grade 5 (*n* = 16)	*p*
Baseline PET/CT findings				0.444
PT only	9 (30)	6 (42)	3 (19)	
PT + LN	6 (20)	3 (22)	3 (19)	
PT + BM	5 (17)	2 (14)	3 (19)	
PT + LN + BM	10 (33)	3 (22)	7 (43)	
Impact on traditional imaging				0.538
Upstaging	5 (17)	2 (14)	3 (19)	
Downstaging	1 (3)	1 (7)	0 (0)	
No change	24 (80)	11 (79)	13 (81)	
Response assessment at 2nd PET/CT scan				0.467
Complete response	2 (7)	2 (14)	0 (0)	
Partial response	24 (80)	10 (72)	14 (88)	
Stable disease	2 (7)	1 (7)	1 (6)	
Disease progression	2 (7)	1 (7)	1 (6)	

BM, bone metastases; LN, metastatic lymph nodes; PSMA, prostate-specific membrane antigen; PT: prostate tumor. Data expressed as counts (percentages).

**Table 4:** [68Ga]Ga-PSMA-11 PET/CT indices before and 3 months after ADT initiation.

Index		Before ADT	After ADT	Reduction Ratio	*p*
Prostate tumor (*n* = 30)					
	SUVmax	30.1 ± 21.4	14.4 ± 17.6	0.49 ± 0.52	<0.001
	PSMA-TV	28.2 ± 34.3	7.6 ± 13.3	0.50 ± 0.94	0.001
	PSMA-TL	346 ± 519	69 ± 159	0.70 ± 0.49	<0.001
Metastatic lymph nodes (*n* = 16)					
	SUVmax	27.2 ± 20.6	13.0 ± 24.6	0.74 ± 0.37	0.001
	PSMA-TV	52.2 ± 114.3	10.1 ± 26.9	0.89 ± 0.19	0.001
	PSMA-TL	671 ± 1494	120 ± 322	0.92 ± 0.14	0.001
Bone metastases (*n* = 15)					
	SUVmax	28.5 ± 22.4	12.0 ± 14.6	0.63 ± 0.34	0.001
	PSMA-TV	180 ± 454	121 ± 341	0.65 ± 0.37	0.001
	PSMA-TL	1202 ± 2886	663 ± 1795	0.73 ± 0.35	0.001
Whole-body (*n* = 30)					
	wbSUVmax	37.1 ± 22.3	19.3 ± 22.3	0.47 ± 0.43	<0.001
	wbPSMA-TV	161 ± 431	73 ± 248	0.54 ± 0.80	<0.001
	wbPSMA-TL	1375 ± 2945	464 ± 1374	0.72 ± 0.37	<0.001

ADT, androgen deprivation therapy; PSMA-TL, prostate-specific membrane antigen—total lesion; PSMA-TV, prostate-specific membrane antigen—total volume; SUVmax, maximum standardized uptake value. Data expressed as means ± standard deviations.

**Table 5:** Comparison of [68Ga]Ga-PSMA-11 PET/CT indices and reduction ratios in different ISUP subgroups.

		Before ADT			After ADT			Reduction Ratio		
Index		G < 5	G = 5	*p*	G < 5	G = 5	*p*	G < 5	G = 5	*p*
Prostate										
	SUVmax	27.83	32.16	1.000	8.79	19.22	0.153	0.61	0.39	0.141
	PSMA-TV	20.35	35.04	0.448	2.81	11.74	0.042	0.51	0.48	0.256
	PSMA-TL	205	469.5	0.637	20.51	110.6	0.038	0.76	0.64	0.239
Metastatic lymph nodes										
	SUVmax	35.18	23.58	0.320	5.38	16.44	0.923	0.80	0.62	0.678
	PSMA-TV	10.81	70.95	0.891	0.38	14.53	0.756	0.86	0.80	0.317
	PSMA-TL	160.80	903.2	0.827	2.29	173.6	0.665	0.91	0.76	0.317
Bone										
	SUVmax	29.30	28.02	0.768	6.36	14.88	0.361	0.67	0.60	0.762
	PSMA-TV	15.60	262.7	0.679	1.78	180.2	0.361	0.70	0.63	0.762
	TL-PSMA	130.73	1737	0.679	8.69	989.7	0.361	0.74	0.72	0.762
Whole-body										
	wbSUVmax	32.40	41.13	0.473	9.87	27.46	0.021	0.60	0.35	0.006
	wbPSMA-TV	29.46	276.5	0.028	3.58	134.3	0.001	0.50	0.58	0.057
	wbPSMA-TL	302.3	2314	0.028	24.43	848.5	<0.001	0.75	0.68	0.057

ADT, androgen deprivation therapy; PSMA-TL, prostate-specific membrane antigen—total lesion; PSMA-TV, prostate-specific membrane antigen—total volume; SUVmax, maximum standardized uptake value. G, ISUP grade, ISUP, International Society of Urological Pathology. Data are expressed as means; significant *P* values are marked in bold.

## Data Availability

The datasets generated and/or analyzed during the current study are available from the corresponding author on reasonable request.

## Supplementary Materials

The following supporting information can be downloaded at: https://www.mdpi.com/article/10.3390/cancers14051329/s1, Table S1: [68Ga]Ga-PSMA-11 PET/CT findings according to different ADT schemes.
