# Supplementary material for: Impact of Three-Month Androgen Deprivation Therapy on [68Ga]Ga-PSMA-11 PET/CT Indices in Men with Advanced Prostate Cancer—Results from a Pilot Prospective Study
Source: Cancers (Basel). 2022 Mar 4;14(5):1329. doi: 10.3390/cancers14051329 (PMC8909781; doi:10.3390/cancers14051329)
Supplement: Supplementary file 1 [file cancers-14-01329-s001.zip › cancers-1563527-supplementary.pdf]

Table S1. [68Ga]Ga-PSMA-11 PET/CT findings according to different ADT schemes.

	First scan			Second scan			Proportional change		
	ADT alone	Combined treatment	*P*	ADT alone	Combined treatment	*P*	ADT alone	Combined treatment	*P*
Total lesions									
SUVmax	30.05	38.80	0.43	10.90	21.34	0.25	0.58	0.44	0.82
wbPSMA-TV	48.02	189.49	0.82	9.23	89.33	0.60	0.81	0.48	0.35
wbTL-PSMA	410.01	1616.52	0.82	54.62	566.28	0.53	0.87	0.68	0.37
Prostate									
SUVmax	27.23	30.87	0.71	9.99	15.44	0.46	0.64	0.45	0.49
PSMA-TV	21.86	29.77	0.82	4.55	8.33	0.53	0.85	0.41	0.30
TL-PSMA	254.29	369.02	0.94	36.17	76.68	0.53	0.90	0.65	0.32
Lymph nodes									
SUVmax	9.75	29.70	0.33	5.34	14.08	1.00	0.66	0.68	1.00
PSMA-TV	1.42	59.40	0.42	0.26	11.51	0.70	0.91	0.81	0.82
TL-PSMA	11.59	765.41	0.50	1.74	136.98	0.60	0.92	0.79	0.82
Bone									
SUVmax	26.14	29.02	0.73	5.83	13.60	0.63	0.73	0.61	0.84
PSMA-TV	51.37	212.56	0.54	9.19	148.58	1.00	0.68	0.65	0.95
TL-PSMA	303.71	1426.07	0.63	35.74	819.40	0.74	0.77	0.72	0.74

ADT alone: use of leuprorelin or goserelin as monotherapy; Combined treatment: combined use of leuprorelin/goserelin/triptorelin with other hormonal agents.
